# Propagated repolarization of simulated action potentials in cardiac muscle and smooth muscle

**DOI:** 10.1186/1742-4682-2-5

**Published:** 2005-02-14

**Authors:** Nicholas Sperelakis, Lakshminarayanan Ramasamy, Bijoy Kalloor

**Affiliations:** 1 Dept. of Molecular & Cellular Physiology University of Cincinnati College of Medicine Cincinnati, OH 45267-0576 USA; 2 Dept. of Electrical Computer Engineering and Computer Science University of Cincinnati College of Engineering Cincinnati, OH 45219 USA

**Keywords:** Propagated Repolarization, Simulated Action Potentials, PSpice simulations, Electric Field mechanism, Cardiac electrophysiology

## Abstract

**Background:**

Propagation of repolarization is a phenomenon that occurs in cardiac muscle. We wanted to test whether this phenomenon would also occur in our model of simulated action potentials (APs) of cardiac muscle (CM) and smooth muscle (SM) generated with the PSpice program.

**Methods:**

A linear chain of 5 cells was used, with intracellular stimulation of cell #1 for the antegrade propagation and of cell #5 for the retrograde propagation. The hyperpolarizing stimulus parameters applied for termination of the AP in cell #5 were varied over a wide range in order to generate strength / duration (S/D) curves. Because it was not possible to insert a second "black box" (voltage-controlled current source) into the basic units representing segments of excitable membrane that would allow the cells to respond to small hyperpolarizing voltages, gap-junction (g.j.) channels had to be inserted between the cells, represented by inserting a resistor (R_gj_) across the four cell junctions.

**Results:**

Application of sufficient hyperpolarizing current to cell #5 to bring its membrane potential (V_m_) to within the range of the sigmoidal curve of the Na^+ ^conductance (CM) or Ca^++ ^conductance (SM) terminated the AP in cell #5 in an all-or-none fashion. If there were no g.j. channels (R_gj _= ∞), then only cell #5 repolarized to its stable resting potential (RP; -80 mV for CM and -55 mV for SM). The positive junctional cleft potential (V_JC_) produced only a small hyperpolarization of cell #4. However, if many g.j. channels were inserted, more hyperpolarizing current was required (for a constant duration) to repolarize cell #5, but repolarization then propagated into cells 4, 3, 2, and 1. When duration of the pulses was varied, a typical S/D curve, characteristic of excitable membranes, was produced. The chronaxie measured from the S/D curve was about 1.0 ms, similar to that obtained for muscle membranes.

**Conclusions:**

These experiments demonstrate that normal antegrade propagation of excitation can occur in the complete absence of g.j. channels, and therefore no low-resistance pathways between cells, by the electric field (negative V_JC_) developed in the narrow junctional clefts. Because it was not possible to insert a second black-box into the basic units that would allow the cells to respond to small hyperpolarizing voltages, only cell #5 (the cell injected with hyperpolarizing pulses) repolarized in an all-or-none manner. But addition of many g.j. channels allowed repolarization to propagate in a retrograde direction over all 5 cells.

## Introduction

There are no low-resistance connections between the cells in several different cardiac muscle and smooth muscle preparations [reviewed in refs. [1] and [2]]. In a computer simulation study of propagation in cardiac muscle, it was shown that the electric field (EF) that is generated in the narrow junctional clefts, when the prejunctional membrane fires an action potential (AP), depolarizes the postjunctional membrane to its threshold [3-5]. Propagation by mechanisms not requiring low-resistance connections have also been proposed by others [6-9]. This results in excitation of the postjunctional cell, after a brief junctional delay. The total propagation time consists primarily of the summed junctional delays. This results in a staircase-shaped propagation, the surface sarcolemma of each cell firing almost simultaneously [4]. Propagation has been demonstrated to be discontinuous (or saltatory) in cardiac muscle [10-13]. Fast Na^+ ^channels are localized in the junctional membranes of the intercalated disks of cardiac muscle [5,14,15], a requirement for the EF mechanism to work [1-5].

We recently modeled propagation of APs of cardiac muscle and smooth muscle using the PSpice program for circuit design and analysis [16-18]. Like the mathematical simulation published in 1977 [3] and 1991 [4], the EF developed in the junctional clefts (negative V_JC_) was large and sufficient to allow transfer of excitation to the contiguous cell, without the requirement of gap-junction (g.j.) channels. Propagation of excitation can occur by the EF mechanism alone, even when the excitability of the cells was made low. In connexin-43 (heterozygous) and Cx40 knockout mice, propagation in the heart still occurs, but it is slowed [19-22] as predicted by our PSpice simulation study [18].

The present experiments were carried out to study propagated repolarization in this model of simulated action potentials (APs). Propagation of repolarization is a phenomenon that occurs in cardiac muscle [23]. It has been shown that propagation of vasodilation occurs in the microvasculature [24], and that the endothelial cells are involved in the conduction of hyperpolarization and vasodilation in an artery [25]. Therefore, our hypothesis was that propagated repolarization would also occur in our PSpice model.

## Methods

**Figure 1:**
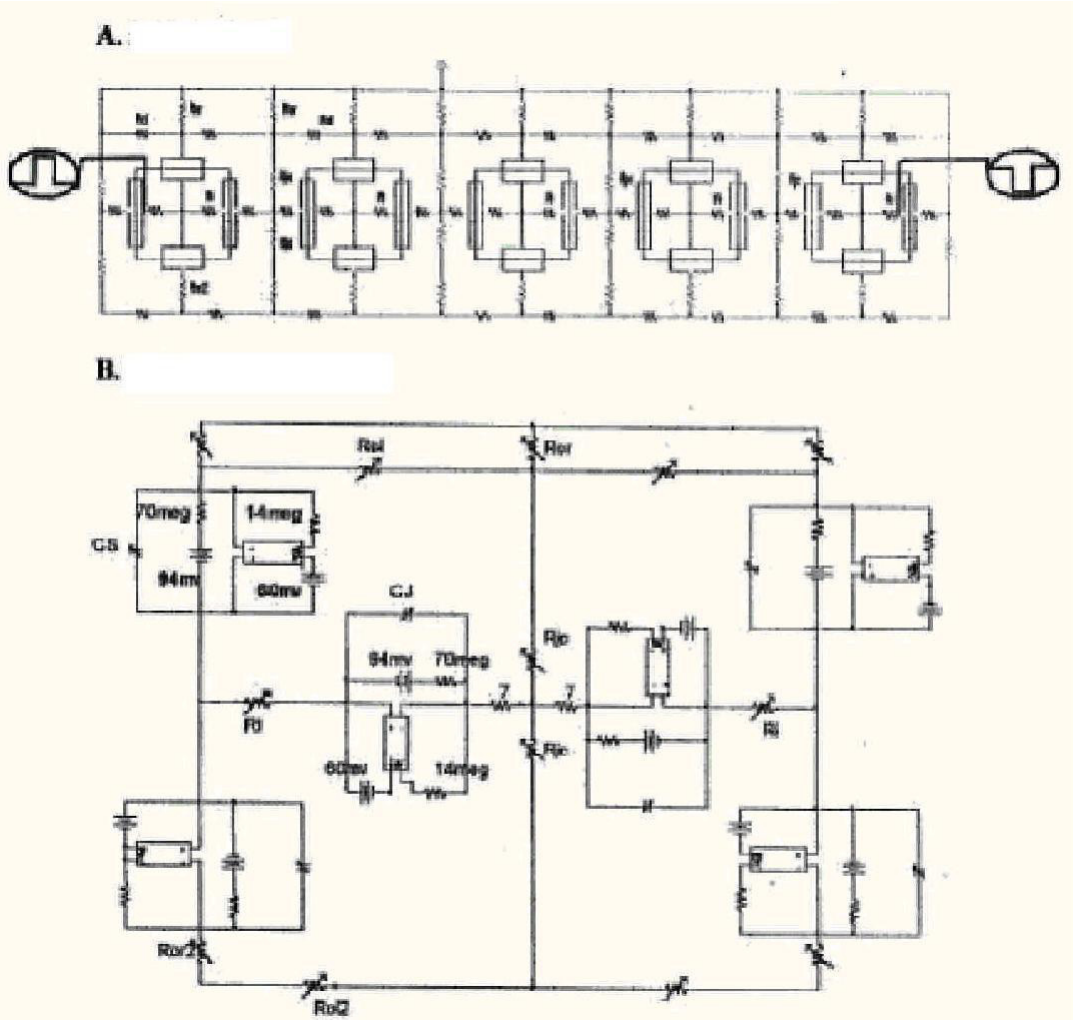
**Cardiac Muscle and Smooth Muscle. **Circuit diagram used for study of propagated repolarization in cardiac muscle and smooth muscle. **A: **5-cell chain. A depolarizing stimulating pulse (I_S1_; 0.50 ms, 0.25 nA) was applied to the inside of the first cell (A1; left side). A hyperpolarizing pulse (I_S2_; variable intensity and duration) was applied to the inside of the fifth cell (A5; right side) a few milliseconds later when the action potentials (APs) initiated by I_S1 _were in their plateau phase (peak overshoot). **B: **Enlarged diagram to show a portion of the circuit for details of the basic units.

The methods used and PSpice program (Cadence Co, Portland) have been described in detail previously, including the circuit [17,18]. In brief, each cell was represented by four basic excitable units, two for the long surface membrane of the cell (one upward-facing and one downward-facing) and one basic unit for each of the two junctional membranes (left end of cell and right end) (Fig 1). The radial (shunt) resistance of the junctional cleft (R_JC_) was placed in the junctions between adjoining cells. The basic units were connected internally by the intracellular longitudinal resistance (r_i_). The basic units were connected externally with the extracellular resistance (R_O_), broken down into a longitudinal component (R_ol_) and a transverse (radial) component (R_or_). R_O _was connected to ground as depicted in Figure 1. The circuit used for each unit was kept as simple as possible, using only those ion channels that set the resting potential (RP) and predominate during the rising phase and plateau phase of the AP.

The myocardial cell was assumed to be a cylinder 150 μm long and 16 μm in diameter, and the smooth muscle cell a cylinder 200 μm long and 5 μm diameter. Since in vascular smooth muscle (VSM), the muscle fibers run in a circular direction, if transverse velocity is calculated, the fiber diameter should be used. The values of the capacitive and the resistive elements in each basic unit were set to reflect the input resistance (ca 20 MΩ) and input capacitance (ca 100 pF) of the individual cells, and the junctional units were prorated, with respect to the surface units, based on relative areas represented. At rest, the resistance of K^+ ^compared to Na^+ ^(cardiac muscle) or Ca^++ ^(smooth muscle) were set to give resting potentials (RPs) of -80 mV for cardiac muscle and -55 mV for smooth muscle. During excitation, the action potentials (APs) overshot to +32 mV and +11 mV, respectively.

Electrical stimulations (I_S1_) were always applied internally to the first cell of the chain (cell A1). Rectangular depolarizing current pulses of 0.25 nA amplitude and 0.50 ms duration were applied. The delay time before the I_S1 _pulse was applied was usually set to 1.0 ms in SM. A second stimulus (I_S2_) that was hyperpolarizing was applied to the inside of the last cell (A5) of the chain when the APs of all 5 cells were in their plateau phase. The intensity and duration of the I_S2 _pulses were varied over a wide range in order to generate strength / duration (S/D) curves.

Because the PSpice program does not have a voltage-dependent resistance (to generate the increase in Na^+ ^or Ca^++ ^conductance during excitation), this function had to be done with a V-controlled current source (our "black-box"). The sigmoidal relationship between conductance and membrane potential (V_M_), over a relatively narrow V_M _range, was mimicked by the black-box. The Na^+ ^or Ca^++ ^current required for excitation had to be calculated for several V_M _values and inserted into the GTABLE function.

Experiments were done with a single chain of 5 cells or 2 cells. There were no gap junctions between the cells of the chain under initial conditions. The presence of gap junction connexons (tunnels) was represented by adding a variable shunt resistance (R_gj_) across each cell-to-cell junction. This resistor connected the inner surface of the prejunctional membrane with the inner surface of the postjunctional membrane. This R_gj _shunt resistance was varied between 10,000 MΩ (1 tunnel), 1000 MΩ (10 tunnels in parallel), 100 MΩ (100 tunnels), 10 MΩ (1,000 tunnels), and 1.0 MΩ (10,000 tunnels). Each tunnel was assumed to have a conductance of 100 pS.

## Results

### A. All-or- None Repolarization of Stimulated Cell A5

**Figure 2:**
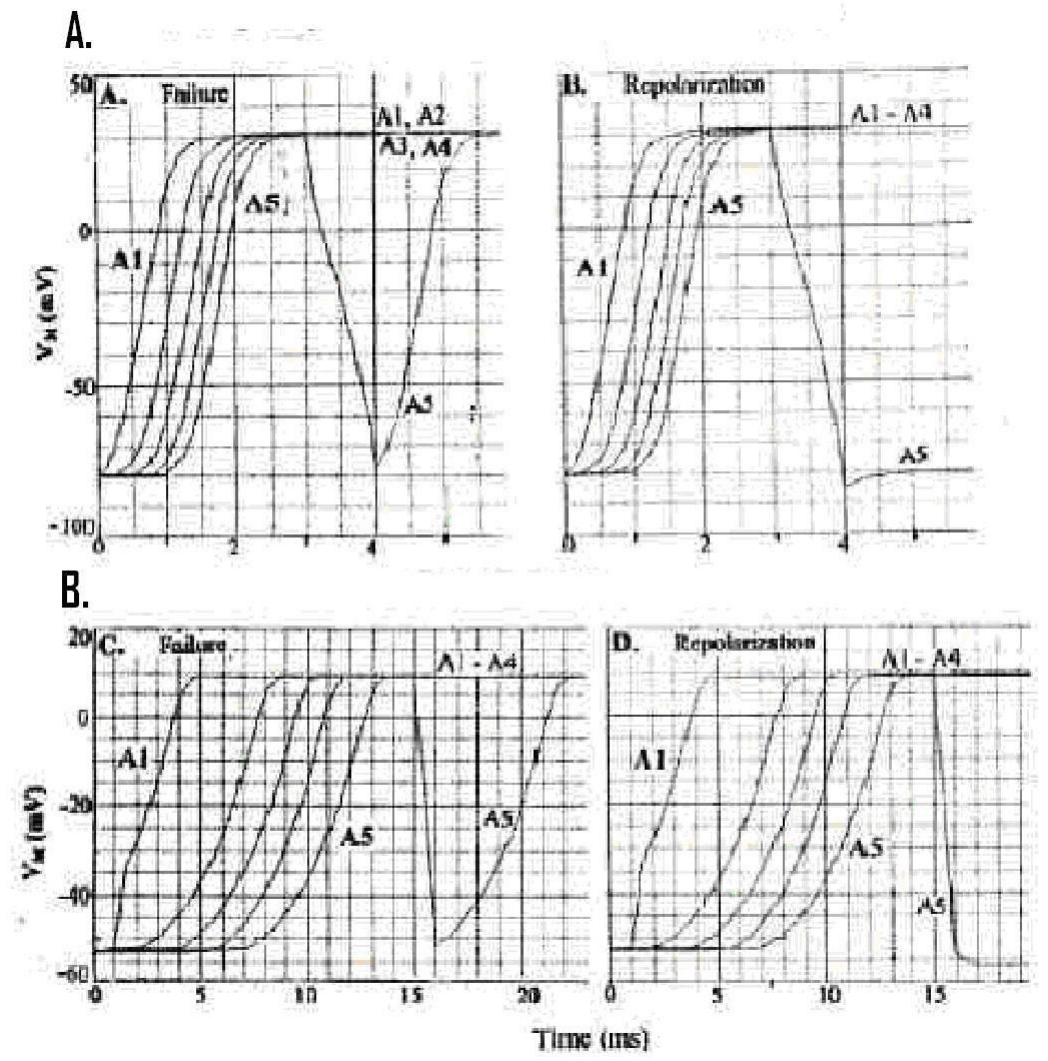
**Sharp Repolarization of Stimulated Cell (A5). **Sharp repolarization of only the last cell (5^th^) of the 5-cell chain when a repolarizing I_S2 _pulse was applied in cardiac muscle (**A-B**) and in smooth muscle (**C-D**). Panels A and C illustrate the records obtained when the applied I_S2 _pulse was just not quite strong enough to produce a permanent repolarization of cell #5. In panels **B **and **D**, the I_S2 _intensity was slightly increased to produce an all-or-none repolarization. The membrane potential of adjacent cell #4 (A4) was only slightly changed when cell A5 underwent a very large change. The velocity of antegrade propagation (θ_a_) was about 54 cm/sec in CM and 8.9 cm/sec in SM under that conditions.

There was a sharp (all-or-none) repolarization of the stimulated cell (A5) of the 5-cell chain in both cardiac muscle (Fig. 2AB) and smooth muscle (Fig. 2CD). As shown, stimulation of cell A1 with a depolarizing current pulse (I_S1_) produced propagation of APs down the chain. At the plateau (peak) of the APs, a repolarizing pulse applied intracelluarly to cell A5, if of sufficient intensity (duration constant), produced a sudden repolarization of only cell A5 (Fig. 2B for cardiac muscle and D for smooth muscle). A slightly lower current intensity failed to produce a stable repolarization of cell A5 (Fig. 2A for cardiac muscle and 2C for smooth muscle). Note that the potential change (repolarizing) produced in neighboring call A4 was very small (< 1 mV). This emphasizes that there are indeed no low-resistance connections between the modeled cells under standard conditions. The hyperpolarizing pulse had to bring the V_m _of cell A5 into the region of the GTABLE's sigmoidal curve. The transient repolarization is in agreement with the biological case [23-25].

### B. Propagation of Repolarization

As indicated in the Methods section, it was not possible to insert a second black-box in the K^+ ^leg of the basic circuit, because the PSpice program became erratic. Therefore, in order to achieve propagation of the repolarization of cell A5 in the retrograde direction, it was necessary to insert gap-junction channels between the cells of the chain (1, 10, 100, 1000, 10000 channels). This corresponded to adding resistive shunts between the cells across the junctions (R_gj_) of 10000, 1000, 100, 10, and 1.0 MΩ (assuming each channel has a conductance of 100 pS).

**Figure 3:**
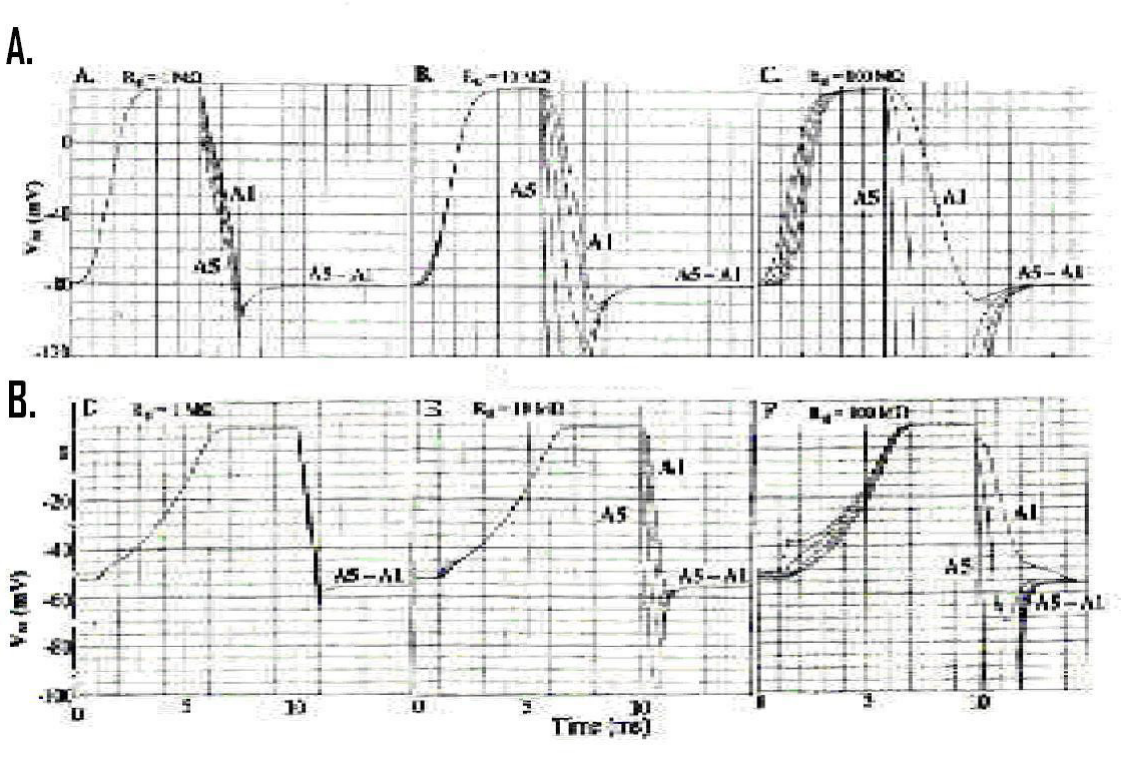
**Insertion of gap junction channels. **Propagation of the repolarization of cell A5 was produced when sufficient gap-junction (g.j) channels were inserted between the cardiac muscle cells and smooth muscle cells. **A: **Record obtained when 10,000 gj-channels were inserted (equivalent to a g.j. resistance (R_gj_) of 1.0 MΩ). Note that the rising phase of the APs from all 5 cells were superimposed, indicating that they all fired simultaneously. Also note that repolarization propagated in a retrograde direction down the 5-cell chain. **B: **1,000 gj-channels inserted (R_gj _of 10 MΩ). Again, the rising phase of the APs of the 5 cells were nearly superimposed. **C: **100 gj-channels (R_gj _of 100 MΩ). With less coupling, the rising phase of the APs of the 5 cells were separated in time. The velocity of propagated repolarization (θ_r_) was further slowed. **D: **R_gj _= 1.0 MΩ(10,000 channels). The rising phase of the APs from all 5 cells were superimposed. Retrograde propagation of repolarization was very fast. **E: **R_gj _= 10 MΩ(1000 channels). The rising phase of the 5 APs were still superimposed, but now the retrograde propagation velocity was slowed. **F: **R_gj_j = 100 MΩ(100 channels). The rising phase of the 5 APs are now separated, indicating velocity of antegrade propagation (θ_a_) was slowed. Velocity of retrograde propagation (θ_r_) was slow.

The results of doing such an experiment are shown in Fig 3 for cardiac muscle (A – C) and for smooth muscle (D – F). When there were many channels (e.g. 10,000 in Fig. 3A and 3D or 1000 in Fig. 3B and 3E), the rising phase of the APs of all 5 cells were superimposed. This means that all 5 cells fired nearly simultaneously, as expected because of the high degree of low-resistance coupling. However, when a repolarizing current pulse was applied to cell A5, its repolarization spread to the neighboring cells. But the other cells did not repolarize simultaneously, as can be seen. Instead, there was a propagation of the repolarization at a certain velocity. This repolarization velocity became slower and slower as the number of channels was decreased. For example, with 100 channels (Fig. 3C and 3F), the propagated repolarization velocity was slower than with 1000 channels (Fig. 3B and 3E) or 10,000 channels (3A and 3D). With only 10 channels, the repolarization did not persist in either cardiac muscle or smooth muscle (not illustrated).

### C. Strength/Duration Curves

**Figure 4:**
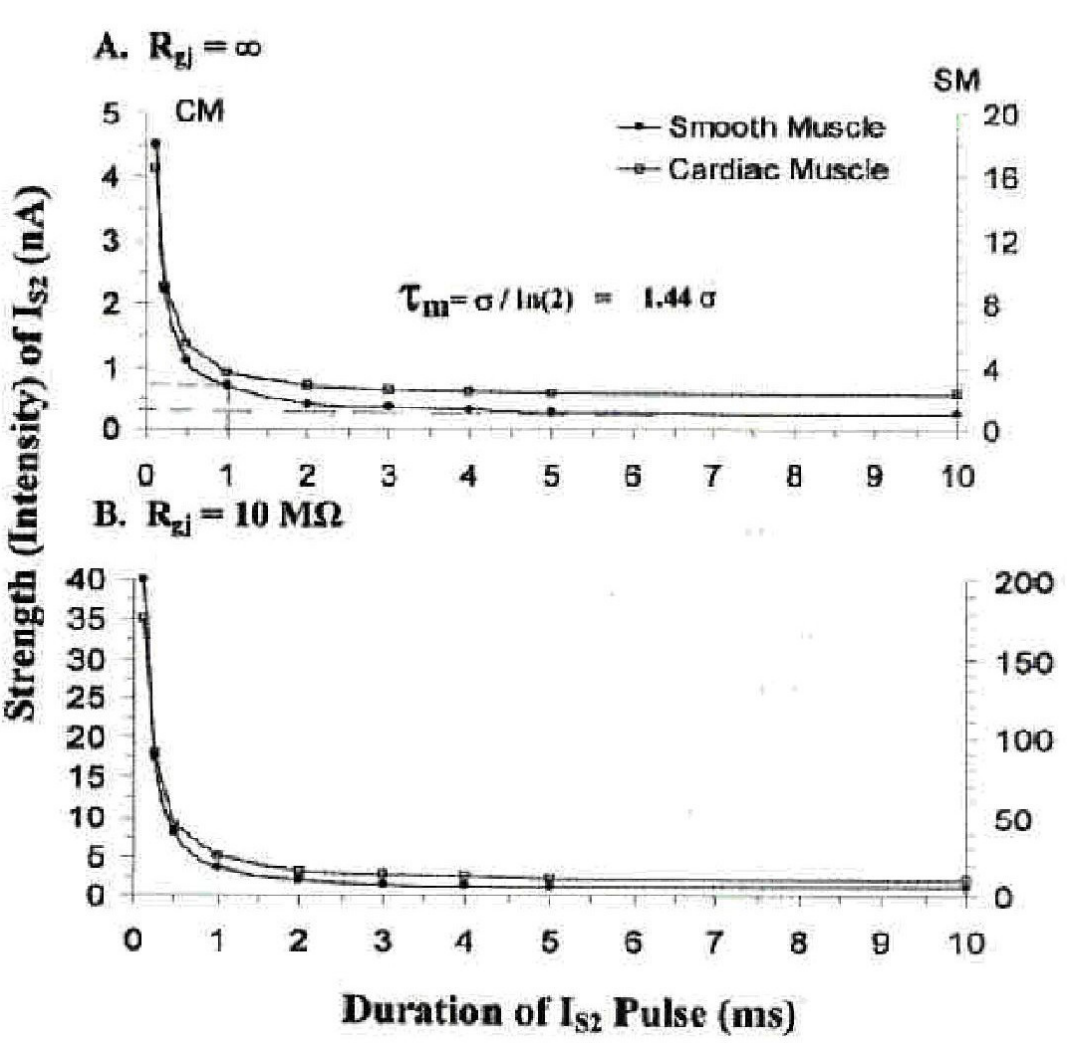
**Strength/Duration Curves. **Strength / duration (S/D) curves for cardiac muscle cells (filled circles) and smooth muscle cells (unfilled circles) (5-cell chains) when R_gj _was ∞ (0 channels) (**A**) or when R_gj _was 10 MΩ(1000 channels) (**B**). The S / D curves are rectangular hyperbolas. The time (pulse duration) it takes for a current intensity of twice the rheobasic intensity to produce the all-or-none repolarization is the chronaxie (σ) The rheobase is the asymptote of the data points extrapolated back to the ordinate, as shown. The chronaxie was about 1.0 ms, in both panels **A **and **B**. But the absolute current intensity required was about 8–10-fold greater in panel **B **compared to panel **A**. The membrane time constant (τ_m_) is related to the chronaxie (σ) by the equation shown in panel **A**.

The intensity (strength) and duration of the rectangular hyperpolarizing current pulses (I_S2_) applied to cell A5 were varied over a wide range in order to generate strength / duration curves. This was done when R_gj _was infinite (i.e., 0 channels) and when R_gj _was 10 MΩ (1000 channels) for strong coupling. The pulse duration was initially constant at 1.0 ms (near the chronaxie value) and then lowered to 0.5 ms and to 0.25 ms. The current intensity was varied until the sharp endpoint occurred, namely the stable repolarization of all cells in the chain. These results are plotted in Figure 4 for cardiac muscle and smooth muscle. **Panel A **is the strength / duration (S / D) curve for when R_gj _was infinite (0 channels), and **Panel B **is the S/D curve for when R_gj _was 10 MΩ(1000 channels). Note that the I_S2 _intensity was about 8–10-fold greater when the cells were well-coupled, because the applied hyperpolarizing current had to spread to all 5 cells of the chain. Regardless, the chronaxie values were about the same (ca. 1.0 ms).

## Discussion

In principle, the addition of a second black-box into the K^+ ^leg of the basic circuit would allow the cell to repolarize in an all-or-none fashion to small repolarizing currents. When this was attempted, the program behaved erratically. So in the absence of g.j. channels, only the cell (A5) injected with repolarizing current (I_S2_) was able to repolarize in an all-or-none manner. The neighboring cell (A4) exhibited only a slight repolarization of <1 mV when cell A5 had repolarized completely back to the RP (-80 mV for CM and -55 mV for SM). This fact emphasized that there were no low-resistance connections between the cells under our initial conditions.

However, addition of 10,000, 1,000, or 100 g.j channels (corresponding to R_gj _values of 1.0, 10, and 100 MΩ) did allow propagation of repolarization to occur. The borderline value was 10 g.j. channels (1000 MΩ R_gj_), e.g., repolarization propagated part-way down the chain in SM and almost succeeded in CM. Of course, inserting the g.j. channels required that the I_S2 _repolarizing current applied be much greater. This is because the I_S2 _current had to spread down the entire chain, with the threshold current required to cause all cells to repolarize being determined by sufficient current entering distal cell A1 to repolarize it to the GTABLE sigmoidal region. Thus the proximal cells, like A5 and A4, became hyperpolarized beyond the level required for their repolarization.

The repolarizing I_S2 _current intensity required for the all-or-none repolarization was lower when the rectangular pulse duration was increased. This was true for both when only the injected cell A5 was repolarized (R_gj _= ∞) and when all 5 cells repolarized (R_gj _of 1.0, 10, and 100 MΩ). Plots of current intensity (ordinate) versus current duration (abscissa) gave the typical hyperbolic strength/ duration curve for excitable membranes. The chronaxie values were about 1.0 ms, for which a time constant τ_m _of about 1.44 ms could be calculated. The S/D curves for the two conditions (R_gj _= ∞ and R_gj _= 10 MΩ) show that the current intensity required was about 8–10-fold greater when there were many gj-channels, in both CM and SM.

The calculated velocity for propagated repolarization (θ_r_) varied with the number of gj-channels (Table 1), as expected. The more channels, the faster the velocity. For cardiac muscle, the θ_r _was about 200 cm/s in the very well coupled case (10,000 channels) and about 50 cm/s in the less coupled case (100 channels) (Table 1). In all cases, the velocity for propagated repolarization (θ_r_) was much lower than the velocity for antegrade propagation (θ_a_.). In the 2-cell chain, the calculated velocities of propagated repolarization were similar to those for the 5-cell chain (Table 1).

**Table 1:** Calculated velocity of retrograde propagation (θ_r_) as compared to that for antegrade propagation (θ_a_) for cardiac muscle (CM) and smooth muscle (SM).

		2-Cell Chain (Cardiac)		5-Cell Chain (cm/sec)			
		
No. of GJ-Channels	Rgj (MΩ)	Threshold* (nA)	θ_r _(cm/s)	CM		SM	
				
				θ_r_	θ_a_	θ_r_	θ_a_
0	∞	#	#	##	32	##	3.7
1	10,000	#	#	##	38	##	6.8
10	1,000	95.4	7.1	##	55	##	13
100	100	15.8	30	50	115	73	42
1000	10	5.4	75	86	550	114	820
10,000	1	5.6	750	200	3000	400	3600

* Pulse duration was held constant at 1.0 ms.

# Second cell (A1) failed to repolarize.

# # Some cells failed to repolarize.

Velocities of antegrade propagation (θ_a_) were taken from previously published data [17,18].

The present study provides some new and important information about the PSpice simulations. First, it verifies that propagation (orthodromic) can occur in the complete absence of gap-junction channels, as previously reported [3,4,16-18]. Second, it demonstrates for the first time that activation of Na+ (in CM) or Ca++ (in SM) channels is reversible, by bringing V_m _back to the level of the sigmoidal activation curve (GTABLE). Third, it shows for the first time that, in the PSpice model, the membranes exhibit the characteristic strength/duration curves. Fourth, it shows that the PSpice program has some serious limitations.

In summary, because of technical difficulties with the PSpice program, it was necessary to insert gj-channels in order to produce propagation of repolarization. Otherwise, only the modeled cell injected (A5) with the repolarizing I_S2 _current was able to repolarize. Since the potential change in the neighboring cell was only about 1 mV or less, this emphasizes that there were no low-resistance connections between the simulated cells under initial conditions. Propagation in the orthodromic direction occurs by the electric field (EF) discussed in previous papers (1–4, 16–18). The repolarizing I_S2 _current gave S/D plots that were typical rectangular hyperbolic curves for excitable membranes, with chronaxie values of about 1.0 ms, both for CM and SM. The calculated velocity for propagated repolarization was greater when the number of gj-channels was increased. The antidromic (retrograde) propagation velocity was usually considerably slower than the orthodromic (antegrade) propagation velocity for depolarization. The present findings do not necessarily imply that, in biological tissue, gap junctions are required for propagated repolarization to occur.
